# Repeatability, Reproducibility, and Observer Variability of Cortical T1 Mapping for Renal Tissue Characterization

**DOI:** 10.1002/jmri.29602

**Published:** 2024-10-28

**Authors:** Magdalena Nowak, Markus Henningsson, Tom Davis, Najib Chowdhury, Andrea Dennis, Carolina Fernandes, Helena Thomaides Brears, Matthew D. Robson

**Affiliations:** ^1^ Perspectum Ltd Oxford UK

**Keywords:** kidney, T1, MOLLI, repeatability, reproducibility

## Abstract

**Background:**

The global rise in kidney diseases underscores the need for reliable, noninvasive imaging biomarkers. Among these, renal cortical T1 has shown promise but further technical validation is still required.

**Purpose:**

To evaluate the repeatability, reproducibility, and observer variability of kidney cortical T1 mapping in human volunteers without known renal disease.

**Study Type:**

Prospective.

**Subjects:**

Three cohorts without renal disease: 1) 25 volunteers (median age 38 [interquartile range, IQR: 28–42] years, female N = 11) for scan–rescan assessments on GE 1.5 T and Siemens 1.5 T; 2) 29 volunteers (median age 29 [IQR: 24–40] years, female N = 15) for scan–rescan assessments on Siemens 3 T; and 3) 16 volunteers (median age 34 [IQR: 31–42] years, female N = 8) for cross‐scanner reproducibility.

**Field Strength/Sequences:**

1.5 T and 3 T, a modified Look‐Locker imaging (MOLLI) sequence with a balanced steady‐state free precession (bSSFP) readout.

**Assessment:**

Kidney cortical T1 data was acquired on GE 1.5 T scanner, Siemens 1.5 T and 3 T scanners. Within‐scanner repeatability and inter/intra‐observer variability: GE 1.5 T and Siemens 1.5 T, and cross‐scanner manufacturer reproducibility: Siemens 1.5 T–GE 1.5 T.

**Statistical Tests:**

Bland Altman analysis, coefficient of variation (CoV), intra‐class coefficient (ICC), and repeatability coefficient (RC).

**Results:**

Renal cortical T1 mapping showed high repeatability and reliability across scanner field strengths and manufacturers (repeatability: CoV 1.9%–2.8%, ICC 0.79–0.88, pooled RC 73 msec; reproducibility: CoV 3.0%, ICC 0.75, RC 90 msec). The method also showed robust observer variability (CoV 0.6%–1.4%, ICC 0.93–0.98, RC 22–48 msec).

**Data Conclusion:**

Kidney cortical T1 mapping is a highly repeatable and reproducible method across MRI manufacturers, field strengths, and observer conditions.

**Evidence Level:**

2

**Technical Efficacy:**

Stage 2

The rising global incidence of acute and chronic kidney diseases (CKD) across both developed and developing countries poses a substantial socio‐economic burden on healthcare systems worldwide.^1^, ^2^, ^3^ The development of better diagnostic and prognostic tools is a key strategy to address these challenges,^3^ and can improve clinical trial endpoints and optimize patient selection. Current clinical biomarkers, such as glomerular filtration rate (GFR) and proteinuria often do not provide insights into the etiology of underlying kidney disease, as they tend to deteriorate only late in the disease course, and do not allow individual patient stratification for prognostic or therapeutic decision‐making.^4^, ^5^ Kidney biopsy is the only standard method to assess renal microstructure but carries risks of serious complications and is susceptible to sampling bias.^6^ MRI techniques, such as T1 mapping, have emerged as a promising, noninvasive approach to assess pathophysiological processes and do not require ionizing radiation and frequently not even contrast agents.^7^, ^8^

Renal cortical T1 mapping has been shown to reflect the degree of tissue fibrosis,^9^, ^10^ predict graft functionality after kidney transplantation,^11^, ^12^ and is significantly increased in CKD.^13^, ^14^ The influence of technical factors on the measurement of T1 values, including field strength, scanner manufacturer and model, observer variability, and patient‐specific considerations, has been well‐documented both within the renal domain and in other anatomical areas.^13^, ^15^, ^16^, ^17^, ^18^ Despite the acknowledged impact of these factors, a considerable gap still exists in providing comprehensive evidence for the technical validation of renal T1 mapping. The task force on technical recommendations for clinical renal MRI, established by the PARENCHIMA,^3^ has emphasized the importance of technical validation efforts, which are needed to support reliable multi‐site clinical studies. Here, we aim to assess the key components of technical validation for renal cortical T1 mapping, focusing on scan–rescan repeatability, inter‐ and intra‐observer variability, and cross‐scanner reproducibility in volunteers without renal disease.

## Materials and Methods

### Study Population and Design

The data were obtained from volunteers in four prospective studies: the technical development project (TECHDEV), the repeatability and reproducibility of multiparametric MRI study (NCT03743272), the COVERSCAN study (NCT04369807), and the MORIS study (NCT04137705). The studies were conducted following ethical standards and received appropriate approvals from local Research Ethics Committees. Inclusion criteria were: male and female subjects aged over 18 years, healthy or with type 2 diabetes, and with ability to understand and sign a written informed consent form. Exclusion criteria included the presence of any MRI contraindications (including pregnancy, extensive tattoos, pacemaker, shrapnel injury, severe claustrophobia). All participants gave their informed consent to participate.

**FIGURE 1:**
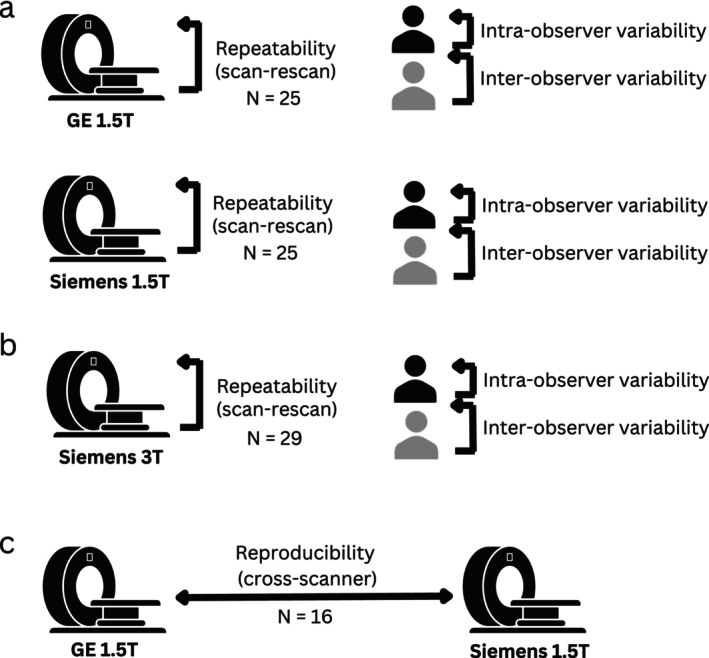
Study design. Repeatability and intra/inter‐observer variability of cortical T1 were assessed on (**a**) GE and Siemens at 1.5 T, (**b**) Siemens at 3 T; (**c**) cross‐scanner reproducibility was assessed between GE 1.5 T and Siemens 1.5 T.

The study is structured into three parts: 1) scan–rescan repeatability and inter/intra‐observer variability study on GE 1.5 T and Siemens 1.5 T; 2) scan–rescan repeatability and inter/intra‐observer variability study on Siemens 3 T; and 3) cross‐scanner (GE 1.5 T–Siemens 1.5 T) reproducibility study (Fig. 1). The data from 25, 29, and 16 participants were included in parts (1), (2), and (3), respectively. Participants underwent either four multiparametric MRI examinations (scan–rescan on both GE 1.5 T and Siemens 1.5 T) or two MRI scans (scan–rescan on Siemens 3 T or reproducibility assessment on GE 1.5 T–Siemens 1.5 T) (Fig. 1). The scan–rescan repeatability experiments involved scans performed under the same conditions, with a maximum interval of approximately 30 minutes between the scans, during which the participant was taken out of the scanner. The cross‐scanner reproducibility study involved scanning participants at the same time during the day on both a Siemens 1.5 T scanner and a GE 1.5 T scanner, 1 week apart, with the order of scanners randomized for each participant.

All authors of this study are employees of Perspectum Ltd., the company that developed the T1 mapping software used for the analysis.

### Data Acquisition

Data acquisitions were performed at Oxford Centre for Clinical Magnetic Resonance Research or at Perspectum Ltd., Oxford, UK. Participants were instructed not to eat, drink, or take medication for 3 hours before their scan time. All participants were scanned using GE (Signa Voyager 1.5 T Px26) and/or Siemens scanners (Aera Syngo MR E11 1.5 T and Prisma Syngo E11C 3 T). Participants were positioned supine for all scans. Phased array coils were used (Signa Voyager 1.5 T Px26: 16‐channel body coil with 32‐channel spine coil; Aera Syngo MR E11 1.5 T: 6‐channel body coil with 24‐channel spine coil; Prisma Syngo E11C 3 T: 18‐channel body coil with 32‐channel spine coil).

A modified Look‐Locker inversion recovery sequence (MOLLI) with a balanced steady‐state free precession (bSSFP) readout was used for all T1 mapping experiments consistent with the consensus‐based technical recommendations^19^ and matched closely between the scanner manufacturers (Table 1). MOLLI data were acquired during breath‐holds with simulated cardiac gating (60 bpm). In all cases, simulated ECG triggering was used at 60 beats/minute. T1 maps were generated using FDA‐cleared and UKCA‐marked software (CoverScan MD v1, Perspectum Ltd.). Data were fitted with a mono‐exponential recovery model with a three‐parameter fit (T1*, inversion efficiency and baseline magnetization), followed by the standard Look‐Locker correction,^20^ in accordance with consensus guidelines.^19^ Complex data (magnitude and phase) were used for fitting of Siemens MOLLI T1 while magnitude data were used for GE MOLLI T1. Unlike vendor‐provided T1 maps, which do not account for variations between MRI systems, CoverScan software is independent of the variations due to scanner model and achieves validated cross‐scanner standardization.^15^ This facilitates comparison of T1 value across different MRI systems and allows the same reference ranges to be used on GE 1.5 T and Siemens 1.5 T scanners. At 3 T, the T1 values are different to those at 1.5 T and CoverScan v1 does not standardize kidney T1 between field strengths.

**TABLE 1:** T1 Mapping Acquisition Parameters for GE at 1.5 T, Siemens at 1.5 T, and Siemens at 3 T

Scanner Manufacturer	MOLLI Scheme	TE (msec)	TR (msec)	Flip Angle (°)	BW (Hz/Pixel)	Acq Res (mm^2^)	FOV (mm)	Slice Thickness (mm)	Parallel Imaging	Data Type
Siemens 3 T	5(3)3	1.09	2.63	35	965	1.6 × 1.6	300 × 300	8	GRAPPA 2	Complex
Siemens 1.5 T	5(3)3	1.49	3.73	35	500	1.6 × 1.6	300 × 300	8	GRAPPA 2	Complex
GE 1.5 T	5(3s)3	1.50	3.41	35	781	1.6 × 1.6	300 × 300	8	ASSET 2	Magnitude

Acq res = acquisition resolution; BW = bandwidth; FOV = field of view; MOLLI = modified Look‐Locker imaging; TR = repetition time; TE = echo time.

### Data Processing

All analyses were performed by two medical imaging analysts employed by Perspectum Ltd. Both analysts had a minimum of 1 year experience in quantitative abdominal MRI analysis and underwent training on the CoverScan software. The analysts, referred to as observers, were fully blinded as to the scanner manufacturer, scanner model, and field strength. For each acquisition, 10 circular regions‐of‐interest (ROI) of 3‐mm diameter in the renal cortex of the T1 maps for each kidney were selected (Fig. S1 in the Supplemental Material).^21^ ROIs were positioned carefully to avoid partial volume effects from adjacent tissue interfaces, such as renal sinus fat or perirenal fat. Scan–rescan repeatability was evaluated as the difference between two repeats (Scan 1 and Scan 2) under the same measurement conditions and analyzed by the same observer. To assess inter‐observer variability, two internal observers processed the same cases separately. To assess intra‐observer variability, one internal observer processed the same set of cases twice (within 2 weeks), with the anonymized cases presented in a random order. To determine cross‐scanner reproducibility, the variability across the repeated kidney cortical T1 measurements under different conditions, i.e., different scanner manufacturers, was measured.

### Statistical Analysis

The following were performed for scan–rescan repeatability, for intra‐ and inter‐observer variability, and for cross‐scanner reproducibility: Bland Altman analysis (bias and 95% limits of agreement [LoA]),^22^, ^23^ coefficient of variation (CoV), intra‐class coefficient (ICC, two‐way mixed effects, absolute agreement, single measurement), and repeatability coefficient (RC). The RC, an estimate of the maximum difference that only 5% of measurement pairs will exceed, was calculated according to standard formulae.^24^, ^25^ In addition, the pooled RC across both GE at 1.5 T and Siemens at 1.5 T scanners was determined in the scan–rescan repeatability experiment. Agreement was classified as follows: ICC >0.9, excellent; 0.9–0.75, good; 0.75–0.5, moderate; <0.5, poor.^26^

The required sample size for the performance testing of kidney cortical T1 was calculated based on Bland Altman estimates, following the methodology outlined by Lu et al,^27^ with type I and type II errors set to 0.05 and 0.20, respectively. Due to a restriction of pilot data to a single scanner (Siemens 1.5 T), uniform performance across all scanners assessed was assumed for the sample size calculation. An observed nonsignificant bias in the pilot study led to an assumed mean difference of 0 msec for cortical T1 repeatability, reproducibility, and observer assessments. The within‐subject standard deviation estimate was also derived from pilot data, with delta bounds set for each experiment according to previously posited limits for clinical utility.^12^, ^28^ The calculations for minimal sample size yielded N = 15 for the left kidney and N = 13 for the right kidney, with the highest N value across all experiments being selected. The final sample sizes adopted in the analyses were met or exceeded those reported in similar studies on kidney cortical T1.^13^, ^16^, ^17^, ^29^ The cortical T1 value for each kidney was determined by first calculating the median T1 for each of the 10 individual ROIs, followed by computing the median of these values. Since the number of observations included in each statistical analysis depends on the results of the quality control, the number of kidneys (Tables 3 and 4) or subjects (Tables S1 and S2 in the Supplemental Material) are specified for each analysis. The analyses were performed and reported for all kidneys (on a per‐kidney basis) and separately for right and left kidney (on a per‐subject basis). Data are reported as mean (standard deviation [SD]) or median (interquartile range [IQR]), where appropriate. A threshold of significance was set at *P* < 0.05 for all statistical tests, with values below this threshold considered statistically significant. All analyses were performed in R (version 4.3, R Project for Statistical Computing, Vienna, Austria).^30^

## Results

A total of 70 participants were included in the analyses. Twenty‐five participants (male = 14, median age = 38 years) were included in the scan–rescan repeatability and operator variability assessments on GE and Siemens 1.5 T scanners, and 29 participants (male = 14, median age = 29 years) were included in the repeatability and operator variability assessments on Siemens 3 T scanner. For cross‐scanner reproducibility, 16 participants (male = 8, median age = 34 years) underwent MRI exams at GE and Siemens 1.5 T scanners. None of the recruited participants reported having renal disease, while nine participants had type 2 diabetes. Details of demographics are reported in Table 2.

**TABLE 2:** Characteristics of the Study Participants

	Scan–Rescan Repeatability 1^a^	Scan–Rescan Repeatability 2^a^	Cross‐Scanner Reproducibility
Scanner	GE 1.5 T, Siemens 1.5 T	Siemens 3 T	GE 1.5 T–Siemens 1.5 T
Total N	25	29	16
Sex			
Male (N)	14	14	8
Female (N)	11	15	8
Age (years)^b^	38 (28–42)	29 (24–40)	34 (31–42)
BMI (kg/m^2^)^c^	25 (5)	25 (6)	24 (2)
Patients with T2D (N)	5	4	0

BMI = body mass index; T2D = type 2 diabetes.

^a^ Observer variability assessments were also performed.

^b^ Age is expressed as median (interquartile range [IQR]).

^c^ BMI is expressed as mean (standard deviation [SD]).

### Mean Cortical T1 Values Across Different Scanner Field Strengths and Manufacturers

The overall mean cortical T1 values ranged from: 1) 1091 msec to 1113 msec for GE 1.5 T, 2) 1048 msec to 1071 msec for Siemens 1.5 T, and 3) 1356 msec to 1387 msec for Siemens 3 T (Tables 3 and 4; separate values for the left and right kidneys are provided in Tables S1 and S2 in the Supplemental Material).

**TABLE 3:** Summary of Scan–Rescan Repeatability and Cross‐Scanner Reproducibility Results for Kidney Cortical T1

N (Kidneys)	Scanner Manufacturer	Field Strength	Scan 1 (msec)	Scan 2 (msec)	Bias (msec)	Lower LoA (msec)	Upper LoA (msec)	CoV (%)	RC (msec)	ICC
			Mean (SD)	Mean (SD)						
Scan–rescan repeatability										
36	GE	1.5 T	1091 (61)	1105 (71)	14.4	−67.3	96.0	2.8	85	0.79
42	Siemens	1.5 T	1051 (56)	1065 (62)	14.3	−37.0	65.5	2.0	58	0.88
50	Siemens	3 T	1356 (51)	1373 (63)	16.8	−48.7	82.3	1.9	73	0.80
Cross‐scanner reproducibility										
32	GE	1.5 T	1111 (61)		34.5	−25.1	94.2	3.0	90^a^	0.75
	Siemens			1077 (59)						

CoV = coefficient of variation; ICC = intraclass coefficient; LoA = limits of agreement; RC = repeatability coefficient; SD = standard deviation.

^a^ Data are reproducibility coefficients rather than repeatability coefficients.

**TABLE 4:** Summary of Inter‐ and Intra‐Observer Variability for Kidney Cortical T1

N (Kidneys)	Scanner Manufacturer	Field Strength	Analysis 1^a^ (msec)	Analysis 2^a^ (msec)	Bias (msec)	Lower LoA (msec)	Upper LoA (msec)	CoV (%)	RC (msec)	ICC
			Mean (SD)	Mean (SD)						
Inter‐observer variability										
34	GE	1.5 T	1107 (72)	1113 (74)	5.2	−36.5	46.9	1.4	42	0.96
38	Siemens	1.5 T	1064 (59)	1071 (63)	7.3	−32.4	47.0	1.4	42	0.94
52	Siemens	3 T	1373 (62)	1387 (63)	14.4	−24.7	53.5	1.3	48	0.93
Intra‐observer variability										
38	GE	1.5 T	1093 (60)	1099 (64)	5.7	−35.1	46.6	1.4	42	0.94
46	Siemens	1.5 T	1051 (58)	1048 (64)	−2.7	−39.8	34.4	1.3	37	0.95
50	Siemens	3 T	1356 (51)	1357 (51)	0.6	−21.6	22.8	0.6	22	0.98

CoV = coefficient of variation; ICC = intraclass coefficient; LoA = limits of agreement; RC = repeatability coefficient; SD = standard deviation.

^a^ Analysis 1 and 2 correspond to the first and second measurement taken either by the same observer (intra‐observer) or by different observers (inter‐observer).

### Scan–Rescan Repeatability and Cross‐Scanner Reproducibility

Kidney cortical T1 mapping demonstrated high scan–rescan repeatability across the tested scanner field strengths and manufacturers, with very low variability (CoV 1.9% to 2.8%) and good reliability (ICC 0.79 to 0.88). A minimal bias was observed across all scanners (14.3 msec to 16.8 msec). The lower and upper LoA were slightly narrower for Siemens scanners at both 1.5 T and 3 T compared to GE 1.5 T (Siemens 1.5 T: −37.0 msec to 65.5 msec, Siemens 3 T: −48.7 msec to 82.3 msec, GE 1.5 T: −67.3 msec to 96.0 msec). The RC ranged from 58 msec to 85 msec across the tested scanners, with the pooled RC calculated at 73 msec, corresponding to approximately 6.3% (in percentage difference space).

In assessing cross‐scanner reproducibility between GE 1.5 T and Siemens 1.5 T, kidney cortical T1 mapping showed low variability (CoV 3.0%), good reliability (ICC 0.75), a positive significant bias (34.5 msec) and RC of 90 msec. The LoA values ranged from −25.1 msec to 94.2 msec. Representative images from a single subject in the cross‐scanner reproducibility study, along with the placement of the 10 ROIs, are shown in Fig. S1 in the Supplemental Material.

**FIGURE 2:**
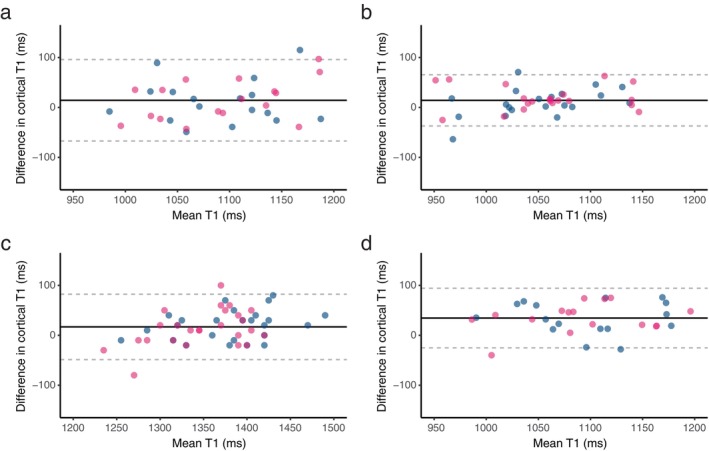
Bland–Altman plots for kidney cortical T1. Scan–rescan repeatability at: (**a**) GE 1.5 T, (**b**) Siemens 1.5 T, (**c**) Siemens 3 T; (**d**) cross‐scanner reproducibility between GE 1.5 T vs. Siemens 1.5 T. Dashed lines show limits of agreement (LoA) of the combined left (blue) and right (pink) kidney data.

Findings are summarized in Table 3 (detailed results for each kidney, analyzed independently, are presented in Table S1 in the Supplemental Material). The bias and LoA are visually supported by Bland–Altman plots (Fig. 2).

### Inter‐ and Intra‐Observer Variability

Both inter and intra‐observer variability measures indicated excellent agreement (ICC 0.93 to 0.96 and ICC 0.94 to 0.98, respectively), with minimal CoV (1.3% to 1.4% for inter‐observer, 0.6% to 1.4% for intra‐observer), negligible bias (from 5.2 msec to 14.4 msec for inter‐observer and −2.7 msec to 5.7 msec for intra‐observer evaluations), and RC between 22 msec and 48 msec. Lower and upper LoA were narrow, demonstrating precise measurements with minimal deviation (inter‐observer LoA ranged from −36.5 msec to 53.5 msec, while intra‐observer LoA ranged from −39.8 msec to 46.6 msec). The findings are summarized in Table 4 (detailed results for each kidney, analyzed independently, are presented in Table S2 in the Supplemental Material). Bland–Altman plots visually confirm the findings, showing the close alignment of cortical T1 measurement values within‐ and across observers (Fig. S2 in the Supplemental Material).

## Discussion

The results presented here show high repeatability and reproducibility of kidney cortical T1 mapping, demonstrating low variability for the scanner field strengths (both 1.5 T and 3 T) and manufacturers (GE and Siemens) tested here. This consistency was also evident in both inter‐ and intra‐observer variability assessments.

The importance of technical validation for renal T1 mapping is underscored by the growing number of studies supporting its clinical utility and validity. Increased T1 has been reported in acute kidney injury, IgA nephropathy and in patients at all stages of CKD.^9^, ^10^, ^13^, ^16^, ^31^ In addition, renal T1 has the potential use for prediction of graft survival/functioning in renal transplant recipients,^12^ and has been shown to perform well (alone or in combination with other biomarkers) in predicting kidney outcomes at 18 months.^32^

The mean cortical T1 values reported here for individuals without renal disease are well within the reference range previously derived using the same methodology: 957 msec–1185 msec (left kidney) and 942 msec–1173 msec (right kidney) for Siemens 1.5 T; 1288 msec–1527 msec (left kidney) and 1278 msec–1516 msec (right kidney) for Siemens 3 T.^33^ As expected, the T1 values were longer at 3 T compared to 1.5 T field strength. Previous investigations addressing technical validity of cortical T1 mapping have predominantly utilized Siemens or Philips scanners at 3 T.^13^, ^16^, ^17^, ^29^ The present study has extended the range to include GE scanners at 1.5 T and Siemens scanners at both 1.5 T and 3 T. The broader range of scanners and field strengths enhances the relevance of cortical T1 mapping for clinical trials by demonstrating technical validity across diverse equipment.

In longitudinal studies where patients undergo repeated scans on the same scanner, within‐scanner (scan–rescan) repeatability is important. Repeatability ensures that diagnostic techniques are consistent over time, allowing for reliable clinical decision‐making and monitoring of disease progression and treatment effects within individual patients. The CoV for scan–rescan repeatability of cortical T1 mapping reported here for volunteers without renal disease (ranging from 1.9% to 2.8%) are comparable to or lower than those reported for commonly used kidney function tests, such as serum cystatin C or creatinine.^34^, ^35^, ^36^ However, considering the distinct physiological properties and clinically relevant dynamic ranges of these methods, comparing their precision performance through CoV alone may not be adequate. Previous MRI studies have reported good scan–rescan repeatability for cortical T1 mapping. The CoV and ICC values reported here for GE 1.5 T and Siemens, at both 1.5 T and 3 T (CoV 1.9% to 2.8% and ICC 0.79 to 0.88), are comparable to results previously reported in healthy volunteers (CoV 0.97% to 5.1%, ICC 0.32 to 0.85)^16^, ^29^, ^37^ and in patients with CKD, diabetic and IgA nephropathy, and in kidney allograft patients (CoV 2.4% to 4.5%; ICC 0.64 to 0.91),^10^, ^13^, ^17^, ^38^ with similar sample sizes across the studies. The repeatability coefficient has not been routinely reported in previous studies on kidney cortical T1. One study performed at 3 T on a Philips scanner reported a scan–rescan repeatability coefficient for cortical T1 of 7.8%,^29^ which is comparable to the results reported in the current study. In the context of longitudinal studies, repeatability coefficient can be considered as the “smallest detectable difference.” This means that in 95% of instances, the magnitude of the repeatability error will not exceed this value. Whether the precision found in this study is sufficient depends on the specific application. The repeatability coefficient can be considered in the context of differences between healthy people and people with different diseases or across various stages of a disease. For example, the repeatability coefficient of 73 msec, as identified in the current study using 1.5 T and 3 T field strength scans, would be sufficient to detect the significant change of ~196 msec in kidney cortical T1 values, reported between mild (eGFR = 60–89 mL/min) and moderate (eGFR = 30–59 mL/min) renal impairment.^12^ In clinical trials, the required effect size threshold for detecting differences, for example in response to treatment, is typically much higher.

In clinical practice and multi‐center clinical studies, reproducibility is the important quality parameter because repeated measurements often occur on different MRI scanners, compromising consistency. The cross‐scanner reproducibility enables consistent results across different scanners and sites, facilitating multi‐center trials and ensuring that diagnostic techniques are widely adoptable and standardized in clinical settings. In addition, reproducibility is vital for facilitating comparisons with reference values that are derived using the same method but from various sources. Here, we have demonstrated a good agreement between repeated T1 measurements across different scanner manufacturers (GE and Siemens) at 1.5 T, with reproducibility coefficient of 90 msec (Table 1). While the bias of 34.5 msec between the GE and Siemens 1.5 T scanners was found to be significant, the mean kidney cortical T1 values were within the normal reference range,^33^ and well below the difference required to differentiate between, for example, mild and moderate renal impairment.^12^

Kidney cortical T1 mapping exhibited robust consistency in inter‐ and intra‐observer variability assessments across the tested scanner field strengths and manufacturers. While studies of inter‐ and intra‐observer variability are scarce, one study reported ICC for intra‐ and inter‐observer variability (combined for healthy and diabetic patients) at 0.89 and 0.83, respectively,^17^ slightly below the ICC range observed in the current study (ICC 0.93–0.98). Another important aspect to consider is a method of ROI selection. The inter‐observer variability has been reported to have lower CoV and higher ICC values when the entire kidney cortex was manually selected, compared to a single ROI approach.^39^ In the present study, the manual selection of 10 ROIs across the whole kidney resulted in CoV (1.3% to 1.4%) and ICC values (ICC 0.93 to 0.96) that align with those obtained from the entire cortex (CoV 1.2%, ICC 0.97).^39^ Additionally, this study found that the intra‐observer variability is comparable to the inter‐observer variability. This could be indicative that our method is less user‐dependent and could achieve similar performance to advanced automated methods.^21^ Understanding the different sources of variability in the measurement may provide guidance for improving the performance of cortical T1. The CoV values for scan–rescan repeatability of cortical T1 were considerably higher than intra‐ and inter‐observer CoV values, suggesting that the variability due to the acquisition rather than the observer dominates the scan–rescan variance. Given that the scan–rescan repeatability experiment was based on consecutive MRI scans (on‐off‐on the scanner), changes in renal physiology are expected to contribute minimally to measurement noise. Instead, thermal noise, scanner calibrations and differences in slice positioning are likely to be the major sources of error. Averaging cortical T1 from repeated measures obtained from separate identical scans could lead to a more stable measurement and better characterization of thermal noise. Similarly, investigations into regional differences in cortical T1 and/or definition of landmarks for a more repeatable slice positioning could further reduce the variability observed.

It is worth noting that T1 mapping values measured with the MOLLI method are dependent on a wide range of factors, including scanner acquisition parameters, eg, the image readout scheme, characteristics of the population studied, and image post‐processing.^16^, ^37^, ^39^ Different T1 mapping readout schemes have been explored in previous renal studies, including spin echo–echo planar imaging (SE‐EPI),^16^ balanced fast field echo (bFFE),^16^ 2D gradient echo planar imaging (2D GE EPI),^29^ and MOLLI.^10^, ^17^ In line with the consensus recommendations,^19^ the present study utilized the MOLLI scheme for T1 mapping. MOLLI is currently the only scheme widely available across MR vendors that can be acquired within a single breath‐hold, making it an appropriate choice for large multi‐center trials comprising multiparametric protocols.

### Limitations

ROIs were manually positioned within the renal cortex, therefore, despite efforts to avoid partial volume effects from adjacent tissues, such as renal sinus fat or perirenal fat, some partial volume effects may still be present. In addition, the current study conducted back‐to‐back MRI scans within a single‐day as part of the scan–rescan repeatability experiment. The short time interval (maximum 30 minutes) between these consecutive assessments may have led to an underestimation of the physiological variability within subjects.^40^ Further, despite our standardization efforts to minimize differences between cortical T1 from different vendors, a significant bias has been observed between paired GE 1.5 T and Siemens 1.5 T data. While this bias is below the minimal detectable difference within a scanner, additional mapping derived from in vivo data may need to be applied to further reduce the bias and improve accuracy. Another limitation of this study is its focus on participants without renal disease. While a small number of individuals with type 2 diabetes were included to provide a broader physiological range, they had no known renal disease, and no conclusions can be drawn from this limited data. Future studies could extend the existing findings to evaluate the accuracy and reliability of quantitative T1 measurements in the presence of renal atrophy and other pathological changes. Further, cortical T1 values at 3 T are substantially longer than those at 1.5 T, due to the known dependency of T1 on magnetic field strength. To report equivalent values across 1.5 T and 3 T scanners, differences due to field strength will need to be incorporated into the mapping functions. Finally, since many previous investigations have included Philips scanners, this study focused on GE and Siemens scanners due to their widespread clinical use. Including additional manufacturers would have increased the study's complexity and required considerably more resources. Future research could incorporate a broader range of manufacturers to enhance the generalizability of the findings.

## Conclusion

The present study demonstrates the reliability of kidney cortical T1 mapping across various MRI scanner field strengths and manufacturers, contributing to the broader goal of standardizing renal MRI procedures. The consistent scan–rescan performance and cross‐scanner reproducibility of this imaging marker support its broader clinical adoption, making it a promising tool for longitudinal monitoring of kidney health in both clinical practice and clinical trial settings.

## Supporting information


**Data S1:** Supporting Information.
