# Supplementary material for: Repeatability, Reproducibility, and Observer Variability of Cortical T1 Mapping for Renal Tissue Characterization
Source: J Magn Reson Imaging. 2024 Oct 28;61(4):1914–22. doi: 10.1002/jmri.29602 (PMC11896918; doi:10.1002/jmri.29602)
Supplement: Supplementary file 1 — Data S1: Supporting Information. [file JMRI-61-1914-s001.docx]

# **Supplementary Tables and Figures**

Figure S1. Representative T1 maps of kidneys (left) along with the placement of ROIs (right) from a single subject from the cross-scanner reproducibility study: a. Siemens 1.5T, cortical T1 (left/right kidney) = 1152/1172 ms; b. GE 1.5T, cortical T1 (left/right kidney) = 1194/1220 ms

| **N** | **Side** | **Scanner manufacturer** | **Field strength** | **Scan 1 (ms) Mean (SD)** | **Scan 2 (ms) Mean (SD)** | **Bias (ms)** | **Lower LoA (ms)** | **Upper LoA (ms)** | **CoV (%)** | **RC (ms)** | **ICC** |
| --- | --- | --- | --- | --- | --- | --- | --- | --- | --- | --- | --- |
|  | **Scan-rescan repeatability** | | | | | | | | | | |
| 18 | Left | GE | 1.5T | 1090 (63) | 1105 (68) | 14.2 | -72.5 | 100.8 | 2.9 | 89 | 0.76 |
| 18 | Right | GE | 1.5T | 1092 (60) | 1106 (75) | 14.5 | -64.4 | 93.4 | 2.6 | 82 | 0.81 |
| 21 | Left | Siemens | 1.5T | 1052 (55) | 1063 (66) | 10.3 | -44.7 | 65.3 | 2.0 | 57 | 0.88 |
| 21 | Right | Siemens | 1.5T | 1049 (59) | 1067 (59) | 18.2 | -29.2 | 65.6 | 2.0 | 58 | 0.88 |
| 25 | Left | Siemens | 3T | 1370 (54) | 1390 (63) | 20.0 | -38.3 | 78.3 | 1.8 | 69 | 0.83 |
| 25 | Right | Siemens | 3T | 1342 (45) | 1356 (60) | 13.6 | -59.1 | 82.3 | 2.0 | 76 | 0.74 |
|  | **Cross-scanner reproducibility** | | | | | | | | | | |
| 16 | Left | GE | 1.5T | 1114 (59) |  | 34.0 | -30.1 | 98.1 | 3.0 | 91^[[1]](#footnote-1)^ | 0.74 |
|  | Right | Siemens |  |  | 1077 (59) | 35.1 | -21.9 | 92.0 | 2.9 | 88^a^ | 0.77 |

Table S1. Summary of scan-rescan repeatability and cross-scanner reproducibility results for kidney cortical T1

^CoV = coefficient of variation, ICC = intraclass coefficient, LoA = limits of agreement, N = number of participants, RC = repeatability coefficient^

Table S2. Summary of inter- and intra-observer variability for kidney cortical T1

| **N** | **Side** | **Scanner manufacturer** | **Field strength** | **Analysis 1^[[2]](#footnote-2)^ (ms) Mean (SD)** | **Analysis 2**^a^ **(ms) Mean (SD)** | **Bias (ms)** | **Lower LoA (ms)** | **Upper LoA (ms)** | **CoV (%)** | **RC (ms)** | **ICC** |
| --- | --- | --- | --- | --- | --- | --- | --- | --- | --- | --- | --- |
|  | **Inter-observer variability** | | | | | | | | | | |
| 17 | Left | GE | 1.5T | 1106 (70) | 1114 (74) | 7.9 | -35.6 | 51.4 | 1.5 | 45 | 0.95 |
| 17 | Right | GE | 1.5T | 1109 (76) | 1112 (76) | 2.5 | -37.9 | 42.8 | 1.3 | 39 | 0.96 |
| 19 | Left | Siemens | 1.5T | 1062 (64) | 1073 (60) | 10.8 | -32.6 | 54.2 | 1.6 | 47 | 0.93 |
| 19 | Right | Siemens | 1.5T | 1067 (56) | 1070 (66) | 3.8 | -31.7 | 39.3 | 1.2 | 35 | 0.96 |
| 26 | Left | Siemens | 3T | 1390 (62) | 1397 (65) | 7.7 | -23.3 | 38.7 | 0.9 | 34 | 0.96 |
| 26 | Right | Siemens | 3T | 1357 (60) | 1378 (60) | 21.2 | -21.2 | 63.5 | 1.5 | 59 | 0.88 |
|  | **Intra-observer variability** | | | | | | | | | | |
| 19 | Left | GE | 1.5T | 1093 (61) | 1103 (70) | 10.4 | -29.4 | 50.2 | 1.4 | 44 | 0.94 |
| 19 | Right | GE | 1.5T | 1094 (59) | 1095 (58) | 1.0 | -39.8 | 41.9 | 1.4 | 40 | 0.94 |
| 23 | Left | Siemens | 1.5T | 1052 (56) | 1045 (63) | 7.2 | -47.7 | 33.3 | 1.5 | 42 | 0.94 |
| 23 | Right | Siemens | 1.5T | 1050 (61) | 1051 (65) | 1.8 | -30.0 | 33.6 | 1.1 | 31 | 0.97 |
| 25 | Left | Siemens | 3T | 1370 (54) | 1372 (53) | 1.6 | -19.3 | 22.5 | 0.6 | 21 | 0.98 |
| 25 | Right | Siemens | 3T | 1342 (45) | 1342 (47) | -0.4 | -24.1 | 23.3 | 0.6 | 23 | 0.97 |

^CoV = coefficient of variation, ICC = intraclass coefficient, LoA = limits of agreement, N = number of participants, RC = repeatability coefficient^


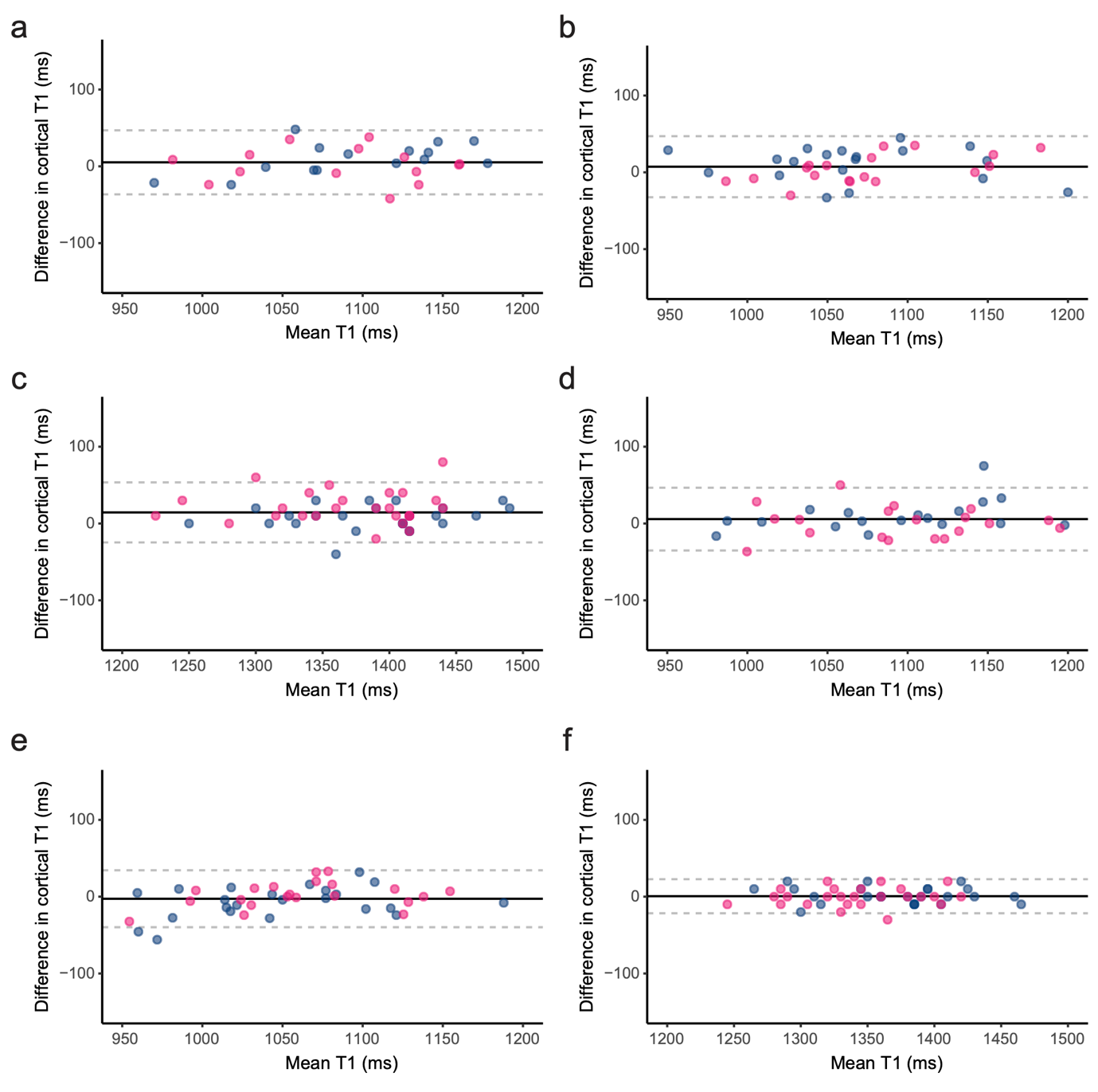

 Figure S2. Bland-Altman plots for kidney cortical T1. Inter-observer variability at: a. GE 1.5T, b. Siemens 1.5T, c. Siemens 3T; and intra-observer variability at: d. GE 1.5T, e. Siemens 1.5T, f. Siemens 3T. Dashed lines show Level of Agreement (LoA) of the combined left (blue) and right (pink) kidney data.

1. Data are reproducibility coefficients rather than repeatability coefficients [↑](#footnote-ref-1)
2. Analysis 1 and 2 correspond to the first and second measurement taken either by the same observer (intra-observer) or by different observers (inter-operator) [↑](#footnote-ref-2)
